# KLK6 Functions as an Oncogene and Unfavorable Prognostic Factor in Bladder Urothelial Carcinoma

**DOI:** 10.1155/2022/3373851

**Published:** 2022-09-22

**Authors:** Kaidong Zhao, Ming Gao, Min Lin

**Affiliations:** ^1^Department of Urology, The Affiliated Hospital of Qingdao University, No. 16 Jiangsu Road, Shinan District, Qingdao, 266003 Shandong Province, China; ^2^Department of Cardiology, The Affiliated Hospital of Qingdao Binhai University, No. 689 Haiya Road, Huangdao District, Qingdao, 266404 Shandong Province, China; ^3^State Key Laboratory of Superamolecular Structure and Materials of Jilin University, No. 2699 Qianjin Avenue, Changchun, 130012 Jilin Province, China

## Abstract

**Background:**

Kallikrein-related peptidase 6 (KLK6) has been substantiated as a diagnostic, prognostic, and therapeutic molecular in several cancer types. In our study, we attempt to explore the biological functions of KLK6 in bladder urothelial carcinoma (BLCA).

**Methods:**

KLK6 gene expression prognostic, gene ontology (GO), Kyoto Encyclopedia of Genes and Genomes (KEGG), gene set enrichment analysis (GSEA), and immune infiltration were analyzed using The Cancer Genome Atlas (TCGA) database. In vitro and in vivo experimental measurements, including CCK8, transwell migration, TUNEL, and nude mouse transplanted tumor model, were used to evaluate the antineoplastic activities of KLK6 loss-of-function.

**Results:**

The combination of bioinformatics analyses and experimental measurements demonstrate that KLK6 expression is aberrantly upregulated in human specimens and cell lines of BLCA. GO and GSEA enrichment analyses exhibited that KLK6 is implicated in the inflammatory response and immune infiltration, suggesting that upregulation of KLK6 may be associated with the progression of BLCA. Knockdown of KLK6 is able to inhibit the growth and migration and trigger apoptosis of RT4 and T24 cells. Moreover, the TCGA database indicates that KLK6 high expression in BLCA patients showed a poorer prognosis than those patients with KLK6 low expression. Univariate and multivariate regression analyses suggest KLK6 as an independent prognostic factor to predict unfavorable OS in patients with BLCA.

**Conclusion:**

KLK6 is an independent prognostic factor and an antitumor target of BLCA. KLK6 expression positively correlates with several immune cells infiltration, indicating that inhibition of KLK6 may contribute to immunotherapy of BLCA.

## 1. Introduction

Bladder urothelial carcinoma (BLCA) is one of the 10 predominant malignancies worldwide and accounts for the majority of bladder cancer (BCa) with more than 90% of diagnosed cases [1]. Global cancer statistics estimate over 573,000 diagnosed individuals and more than 212,000 patients to die from BCa [2]. At present, the pathogenesis of BLCA is complicated, and multiple factors, including heredity, environment, immune infiltration, and metabolism factors, are implicated in this process [3–6]. However, the precise reasons of the development and progression of BLCA are poorly understood. Therefore, it is urgent to explore the molecular mechanisms in BLCA.

KLK6 is a member of serine proteases family and has been substantiated as a biomarker in numerous disorders, such as autoimmunity diseases [7, 8], inflammatory joint disease [7], Alzheimer's disease [9], and brain injury [10]. In addition, KLK6 is linked to cell growth, migration, invasion, poor survival, and chemoradiotherapy resistance of multiple malignant tumors [11–15]. KLK6 is also reported as a potential serum marker for the screening of colorectal cancer [16], uterine serous papillary cancer [17], and breast cancer [18]. In muscle-invasive bladder cancer, KLK6 expression is significantly elevated in deceased patients compared with in living patients, and the multivariable Cox regression model uncovers KLK6 as an independent prognostic gene [19]. Intriguingly, KLK6 may be correlated with immunotherapy in urothelial cancer [20]. Compared with nonresponders to PD-L1 blockade in urothelial cancer, KLK6 expression is significantly upregulated in responders [20].

In our study, we attempted to explore the expression, prognosis, and immune infiltration of KLK6 in BLCA using the TCGA database. Moreover, we also investigated the underlying molecular mechanism and antitumor properties of KLK6 in vitro and in vivo experimental measurements.

## 2. Material and Methods

### 2.1. Screening of Differentially Expressed Genes (DEGs) and Prognostic Factors

DEGs and top 100 prognostic factors were selected using the TCGA database (https://portal.gdc.cancer.gov/). The analytical methods of DEGs and prognostic factors were performed using R software (version 3.6.3) with DESeq2 package (version 1.26.0) [21] and survminer package (version 0.4.9) [22]. The promoter methylation level of KLK6 in BLCA was analyzed by UALCAN database (http://ualcan.path.uab.edu/analysis.html) as described previously [23]. KLK6 expression in tissues was calculated using Human Protein Atlas (HPA) database (https://www.proteinatlas.org/).

### 2.2. GO, KEGG Pathway Enrichment, and GSEA

Top 100 KLK6-related genes were filtrated using the TCGA database with stat package (version 3.6.3). The DAVID on-line database (https://david.ncifcrf.gov/) was implemented to investigate GO, (including BP, biological process, and MF, molecular function) and KEGG pathways based on top 100 KLK6-related genes. Analysis of single gene difference of KLK6 in BLCA was prepared for GSEA using TCGA database with DESeq2 package (version 1.26.0) as described previously [21].

### 2.3. Immune Infiltration

The correlation of KLK6 with Th2 cell enrichment in BLCA was analyzed by GSVA package (version 1.34.0) with ssGSEA algorithm [24, 25]. Th2 cell enrichment generated an unfavorable prognosis in patients with BLCA was analyzed using the Kaplan-Meier Plotter online tool (http://kmplot.com/analysis/index.php?p=background) as described previously [26].

### 2.4. Cell Culture and Small Interfering RNA (siRNA) Transfection

Human BLCA cell lines (RT4, T24, EJ, and BIU87) and normal urinary bladder epithelial cell line SV-HUC-1 were maintained in DMEM with 10% FBS (Thermo Scientific HyClone, Beijing, China), 100 U/ml penicillin, and 100 mg/ml streptomycin in a humidified incubator (Thermo, USA), 5% CO_2_, 95% air atmosphere. Si-NC and si-KLK6 were synthesized by Sangon Biotech (Shanghai, China). Lipofectamine 3000 (Invitrogen) was used to cell transfection according to the manufacturer's instructions. After 48-h transfection with si-NC or si-KLK6, in vitro experimental measurements were performed to evaluate the biological functions of si-KLK6 in BLCA cell lines.

### 2.5. Proliferation, Migration, and Apoptosis Assays In Vitro

CCK-8 kit (Beyotime Institute of Biotechnology, Haimen, China) was used to measure cell proliferation in vitro, and cell viability was analyzed with a SpectraMax M5 ELISA plate reader (Molecular Devices, LLC, Sunnyvale, CA, USA) at 450 nm. Migration was analyzed using the transwell chamber (8 *μ* pore size; Corning Incorporated, Corning, NY, USA). TUNEL kit (Roche) was utilized to analyze cell apoptosis according to the manufacturer's instructions. Nucleus staining with red represents an apoptotic cell, and apoptotic proportion is the ratio of the number of apoptotic cells to total cells.

### 2.6. Western Blot

The primary antibody for KLK6 (cat. no: sc-374564; dilution ratio 1: 5,000) and horseradish peroxidase-conjugated secondary antibody (antirabbit IgG-HRP; cat. no: sc-2357) were obtained from Santa Cruz Biotechnology, Inc. (Dallas, TX, USA). Protein bands were visualized using an enhanced chemiluminescence kit (Thermo Fisher Scientific, Inc.). Signals were analyzed with Quantity One® software version 4.5 (Bio Rad Laboratories, Inc., Hercules, CA, USA). Anti-*β*-actin (cat. no. sc-130065; 1: 2,000; Santa Cruz Biotechnology) was used to as the control antibody.

### 2.7. Animal Model

Five-week-old male BALB/c nude mice were obtained from the Beijing Vital River Laboratory Animal Technology (Beijing, China). After RT4 and T24 cell transfection with si-NC or si-KLK6, cells were subcutaneously implanted into nude mice as described previously [27]. After 4 weeks with cell inoculation, tumor tissues were collected from nude mice.

### 2.8. Statistical Analysis

Data were presented as mean ± standard deviation. The Student's *t*-test, Mann–Whitney *U* test, or Dunn's test was used to analyze two-group differences. Intergroup differences were analyzed by one-way ANOVA. Survival analysis was performed using the log-rank test and univariate and multivariate Cox regression analysis. *P* value less than 0.05 was considered a significant difference.

## 3. Results

### 3.1. Screening of Differentially Expressed Genes (DEGs) to Determine Prognostic Factors in BLCA

**Figure 1:**
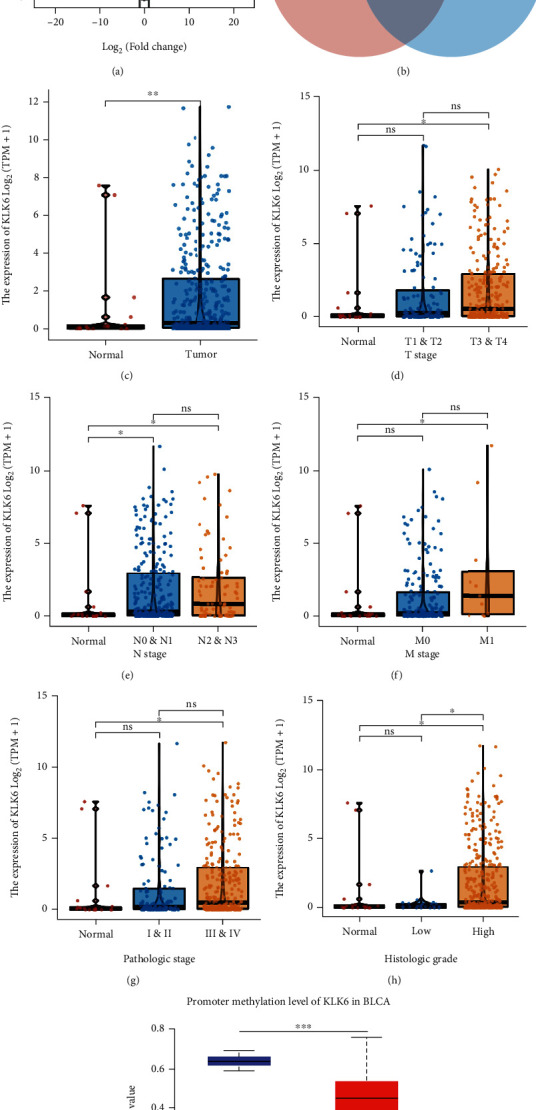
KLK6 expression and the association of KLK6 with clinical parameters. Volcano plot represents the gene expression between BLCA tissues (*n* = 414) and normal tissues (*n* = 19), and DEGs were filtrated using |*Log*2(fold change)| > 1 and Adj.p < 0.05 (a). The Venn diagram represents the number of genes between DEGs and the top100 prognostic factors (b). KLK6 expression was significantly up-regulated in BLCA tissues (*n* = 414) compared with normal tissues (*n* = 19) (c). The association of KLK6 gene expression with clinical parameters, including T stage (d), N stage (e), M stage (f), pathologic stage (g), and histologic stage (h). The promoter methylation level of KLK6 in BLCA was analyzed by UALCAN database (i). ^∗^*p* < 0.05, ^∗∗^*p* < 0.01, ^∗∗∗^*p* < 0.001; ns: no significant.

DEGs (Figure 1(a)) and top 100 prognostic factors (Figure 1(b)) were filtrated using the TCGA database. We found that KLK6 was significantly upregulated and served as a prognostic factor in patients with BLCA (Figures 1(a)–1(c)). Compared with the normal group, the elevation of KLK6 gene expression was exhibited in T3/T4 stage (Figure 1(d)), N0/N1 and N2/N3 stage (Figure 1(e)), M1 stage (Figure 1(f)), III/IV pathologic stage (Figure 1(g)), and high histologic stage (Figure 1(h)), suggesting that KLK6 high expression was associated with advanced clinical classifications. The promoter methylation level of KLK6 in BLCA was analyzed by UALCAN database, and a significant reduction of KLK6 methylation was observed in BLCA patients (*n* = 418) compared with normal subjects (Figure 1(i)), which offers a possible explanation of upregulated KLK6 expression in BLCA patients at the epigenetic mechanism.

### 3.2. KLK6 Expression in Normal Tissues and Pan-Cancer Tissues

**Figure 2:**
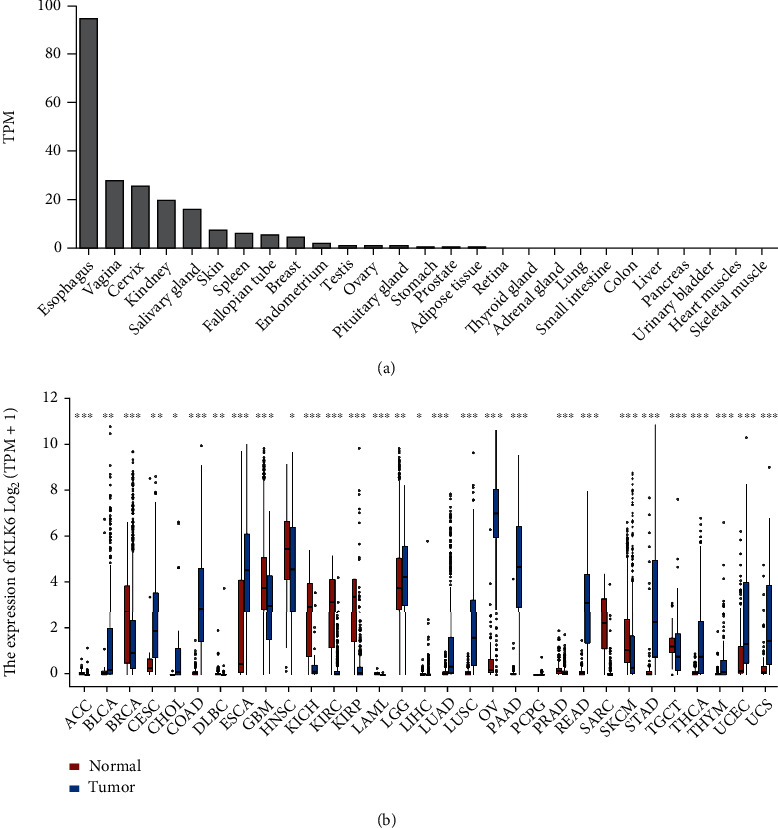
KLK6 expression in normal tissues and Pan-cancer tissues. KLK6 gene expression in normal tissues was analyzed using the HPA database (a). KLK6 gene expression in pan-cancer was evaluated using the TCGA database (b). ^∗^*p* < 0.05, ^∗∗^*p* < 0.01, ^∗∗∗^*p* < 0.001.

KLK6 was abundantly expressed in several tissues or organs, such as the esophagus, vagina, cervix, kidney, salivary gland, skin, spleen, fallopian tube, and breast. However, KLK6 expression has not been observed in the urinary bladder, reflecting that KLK6 may be a tumor-related gene accompanied by the progression of BLCA (Figure 2(a)). As shown in Figure 2(b), KLK6 was significantly decreased in 13 cancer types, and KLK6 was significantly increased in 16 cancer types compared with normal tissues (Figure 2(b)).

### 3.3. KLK6 Is an Independent Prognostic Factor in Patients with BLCA

**Figure 3:**
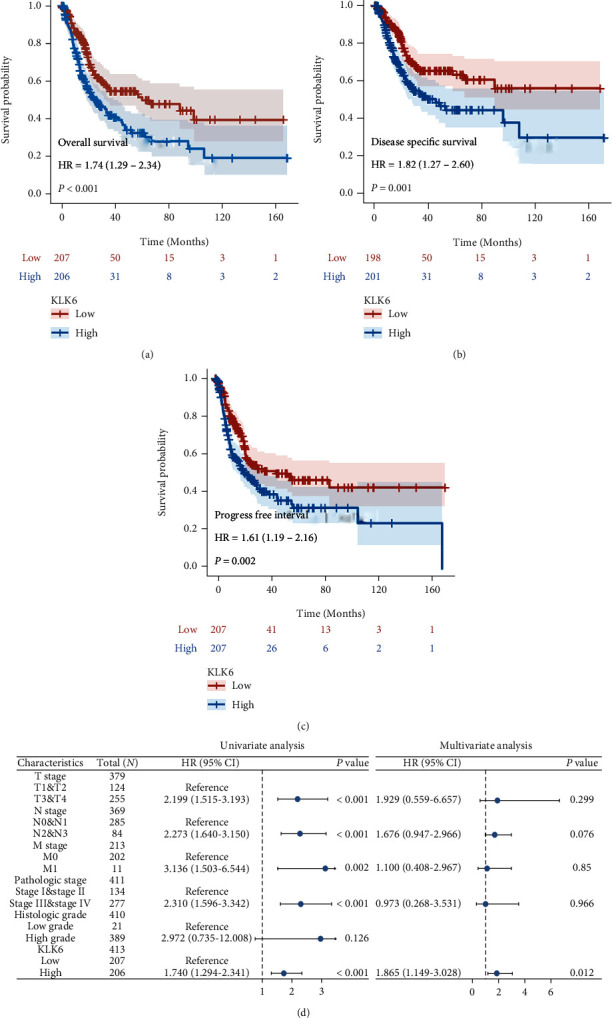
KLK6 is an independent prognostic factor in patients with BLCA. TCGA revealed the association of KLK6 with overall survival (a), disease-specific survival (b), and progress-free interval (c) in patients with BLCA. Both univariate and multivariate regression analyses were implemented to evaluate the risk factors of OS in patients with BLCA (d).

TCGA revealed that KLK6 high expression in BLCA patients showed a shorter overall survival [HR = 1.74 (1.29-2.34), *p* < 0.001; Figure 3(a)], disease-specific survival [HR = 1.82 (1.27-2.60), *p* = 0.001; Figure 3(b)], and progress-free interval [HR = 1.61 (1.19-2.16), *p* = 0.002; Figure 3(c)] than those patients with KLK6 low expression. Both univariate and multivariate regression analyses were implemented to evaluate the risk factors of OS in patients with BLCA. As shown in Figure 3(d), univariate regression analysis shows that advanced T stage, N stage, M stage, pathologic stage, and KLK6 high expression were the risk factors of OS in patients with BLCA. However, multivariate regression analysis indicated KLK6 as an independent prognostic factor to predict unfavorable OS in patients with BLCA. In addition, except LUAD [HR = 1.35 (1.01-1.80), *p* = 0.04], the gene expression of KLK6 had no significant correlation with OS in CESC, CHOL, COAD, ESCA, LGG, LUSC, OV, PAAD, READ, STAD, THCA, THYM, UCEC, and UCS (Supplementary Figure 1).

### 3.4. GO, KEGG, and GSEA of KLK6-Related Genes in BLCA

**Figure 4:**
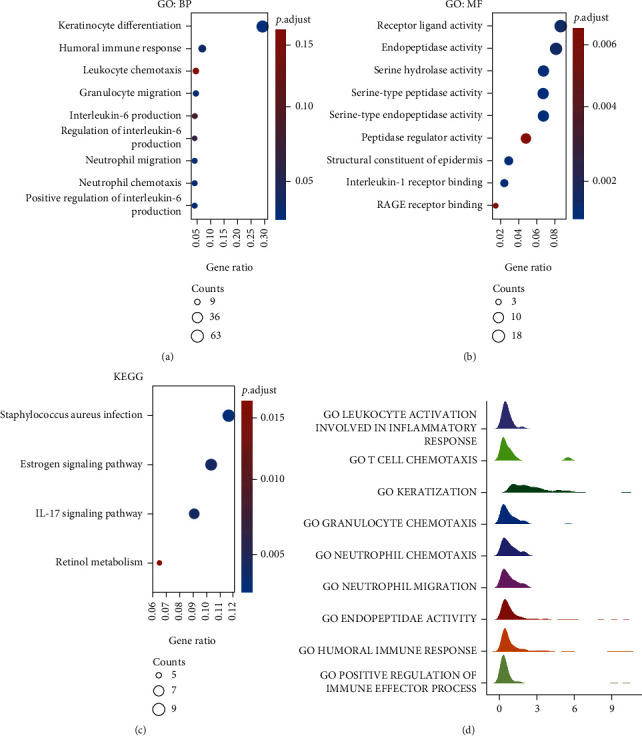
GO, KEGG, and GSEA of KLK6-related genes in BLCA. GO, including BP (a) and MF (b) and KEGG (c) and GSEA (d), revealed that KLK6-related genes were enriched in multiple signaling pathways.

GO, including BP (Figure 4(a)) and MF (Figure 4(b)) and KEGG (Figure 4(c)) and GSEA (Figure 4(d)), revealed that KLK6-related genes were enriched in the biological processes, such as keratinocyte differentiation, humoral immune response, leukocyte chemotaxis, neutrophil migration, and interleukin production (Figure 4(a)); interleukin-1 receptor binding (Figure 4(b)); interleukin-17 signaling pathway (Figure 4(c)); and leukocyte activation involved in the inflammatory response, T cell chemotaxis, and positive regulation of immune effector process (Figure 4(d)). These findings displayed that KLK6-related genes were implicated in the inflammatory response and immune cell enrichment of BLCA.

### 3.5. KLK6 Related with Th2 Cell Enrichment in BLCA

**Figure 5:**
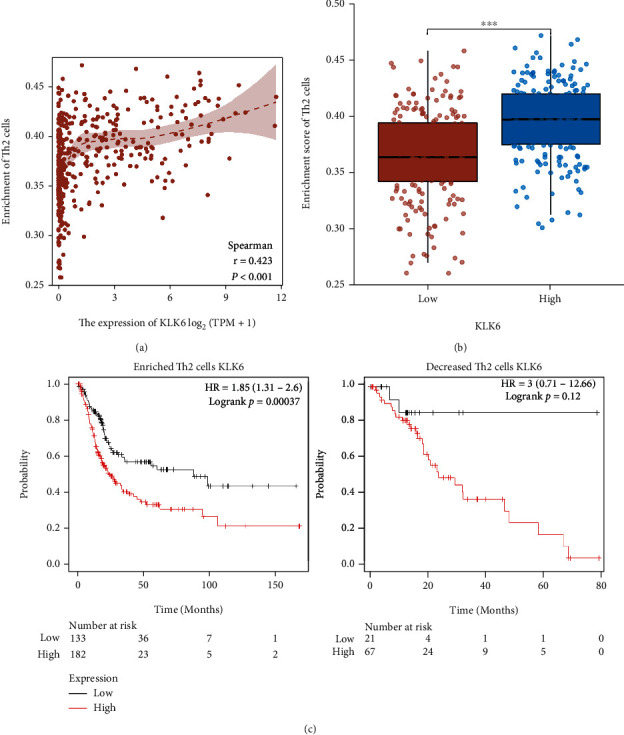
KLK6 related with Th2 cell enrichment in BLCA. The relationship between KLK6 and Th2 cell enrichment in BLCA was implemented using the TCGA database (a). Th2 cell enrichment was associated with upregulation of KLK6 expression in BLCA (b). We analyzed whether Th2 cell enrichment generated an unfavorable prognosis in patients with BLCA using Kaplan-Meier Plotter online tool (c). ^∗∗∗^*p* < 0.001.

To further investigate the relationship between KLK6 and immune cell enrichment in BLCA, the TCGA database was implemented to evaluate KLK6-related immune cells infiltration in the tumor microenvironment of BLCA. As shown in Figure 5(a), the enrichment score of Th2 cells is significantly correlated with KLK6 expression in BLCA (*r* = 0.423; *p* < 0.001). In addition, the high enrichment score of Th2 cells was associated with upregulation of KLK6 expression in BLCA (Figure 5(b)). Next, we analyzed whether Th2 cell enrichment generated an unfavorable prognosis in patients with BLCA. Patients were divided into two groups with enriched and decreased immune cells. As shown in Figure 5(c), KLK6 as an unfavorable prognostic factor was presented in patient with enriched Th2 cells, but not in decreased Th2 cells. These findings exhibited that the enrichment of Th2 cells may be associated with poor prognosis in patients with KLK6 high expression.

### 3.6. Knockdown of KLK6 Suppresses Malignant Phenotypes of BLCA Cells

**Figure 6:**
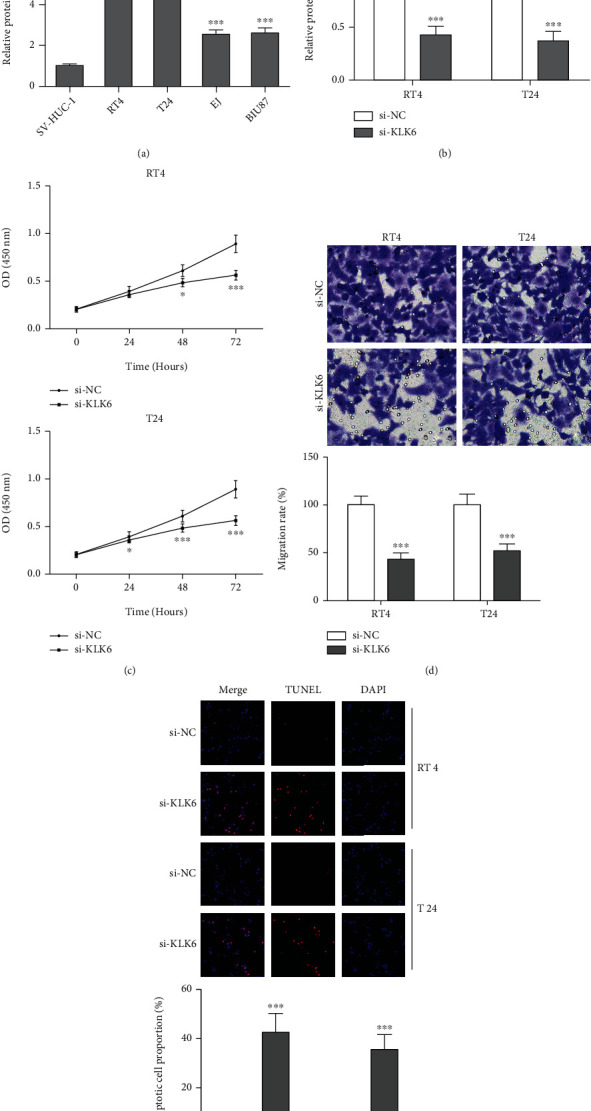
Knockdown of KLK6 suppresses malignant phenotypes of BLCA cells. Upregulation of KLK6 protein expression was detected in four BLCA cells using western blot (a). Transfection with si-KLK6 into RT4 and T24 for 48 h, KLK6 protein expression was detected using western blot (b). After transfection with si-KLK6 or si-NC into RT4 and T24, malignant phenotypes of BLCA cells were evaluated using CCK-8 (c), transwell (d), and TUNEL assays (e).

As shown in Figure 6(a), upregulation of KLK6 protein expression is detected in four BLCA cells (RT4, T24, EJ, and BIU87) compared with normal urinary bladder epithelial cell line SV-HUC-1. Three siRNAs were designed to inhibit the expression of KLK6. As shown in Supplementary Figure 2, si-KLK6 exhibits the most prominent inhibiting effect of cell proliferation, with the inhibition ratio of 53% and 58% in RT4 and T24 cells, respectively. Therefore, si-KLK6 was selected for subsequent cell and animal experiments. Transfection with si-KLK6 into RT4 and T24 led to a significant reduction of KLK6 protein expression compared with the control group (Figure 6(b)). Next, the effects of KLK6 on malignant phenotypes of BLCA cells were evaluated using CCK-8, transwell, and TUNEL assays. Transfection with si-KLK6 into RT4 and T24, cell proliferation (Figure 6(c)), and migration (Figure 6(d)) were markedly retarded, and cell apoptosis (Figure 6(e)) was significantly enhanced compared with the control group.

### 3.7. Knockdown of KLK6 Impedes Cell Growth In Vivo

**Figure 7:**
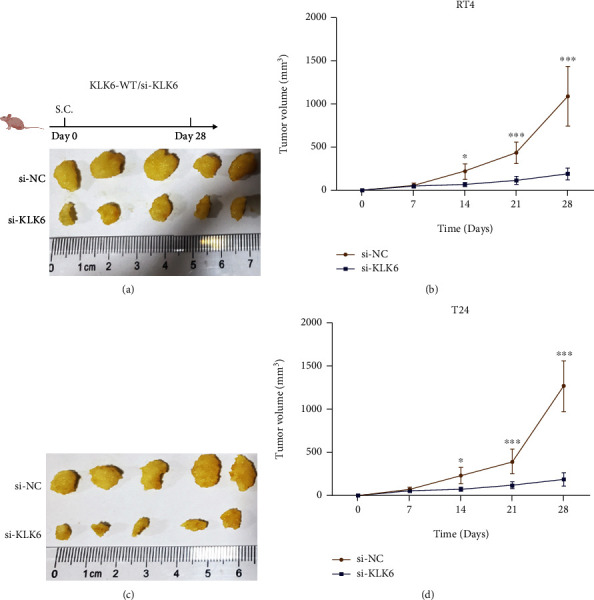
Knockdown of KLK6 impedes cell growth in vivo. The role of si-KLK6 on RT4 (a and b) and T24 (c and d) cell growth within 4 weeks was evaluated using a subcutaneously implanted tumor model. ^∗^*p* < 0.05, ^∗∗∗^*p* < 0.001.

The role of KLK6 on cell growth was evaluated using a subcutaneously implanted tumor model. As shown in Figures 7(a) and 7(b), tumor formation of RT4 cells was faster and larger in the control group than in the si-KLK6 group. Moreover, the same conclusion was observed in the in vivo growth of T24 cells (Figures 7(c) and 7(d)).

## 4. Discussion

In our study, the combination of bioinformatics analyses and experimental measurements demonstrated that KLK6 expression is aberrantly upregulated in human specimens and cell lines of BLCA. GO and GSEA enrichment analyses exhibited that KLK6 is implicated in the inflammatory response and immune infiltration, suggesting that upregulation of KLK6 may be associated with the progression of BLCA. In vitro and in vivo findings represent that knockdown of KLK6 is able to inhibit the growth and migration and trigger apoptosis of RT4 and T24 cells. Moreover, the TCGA database indicates that KLK6 high expression in BLCA patients showed a poorer prognosis than those patients with KLK6 low expression. Univariate and multivariate regression analyses suggest KLK6 as an independent prognostic factor to predict unfavorable OS in patients with BLCA.

KLKs are an important family of 15 proteases to mediate multiple functions via cleaving and activating the members of the G-protein-coupled proteinase-activated receptor family [28]. KLK3 (also known as prostate-specific antigen) is an outstanding member of the KLKs family as a diagnostic marker and therapeutic target of prostate cancer (PCa), and several medicines are approved and undergone clinical research for the treatment of PCa via targeting KLK3 [29, 30]. However, KLK6-related pathways are focused on the preclinical stage and represent the prospective therapeutic targets to prevent tumor progression [31, 32]. Based on TCGA database analysis, KLK6 is significantly upregulated in 16 cancer types. In addition, the elevation of KLK6 expression has been reported in the kinds of literature, such as melanoma [33], ovarian cancer [34], gastric cancer [35], pancreatic ductal adenocarcinoma [36, 37], and colon cancer [38]. In our study, KLK6 is validated as an independent and unfavorable prognostic factor of BLCA. Inhibition of KLK6 by siRNA exhibits the tremendous antineoplastic activities by suppressing tumor growth and inducing cell apoptosis of BLCA cells. Shinoda et al. [39] have reported that knockdown of KLK6 transcript by siRNA significantly reduces the invasion of a bladder carcinoma cell line, suggesting that KLK6 may play a crucial role in promoting cancer cell invasion in bladder tumor. Our findings also revealed that inhibition of KLK6 restrained the malignant phenotypes of bladder cancer cells.

A further finding is that KLK6 high expression is associated with multiple immune cell enrichment, and enriched Th2 cells are correlated with poor prognosis in BLCA patients with KLK6 high expression, suggesting that Th2 cell enrichment may be a risk factor for unfavorable prognosis. Th2 cells can secrete multiple cytokines, such as IL-4, IL-5, IL-10, and IL-13, which are associated with inflammation and tumor-promoting functions [40, 41]. In addition, GO and GSEA analysis revealed that KLK-6 related genes are enriched in the biological process of neutrophils migration and chemotaxis, and a high enrichment score of neutrophils is associated with upregulation of KLK6 expression in BLCA. Neutrophils are the major component of leukocytes to defend infection-related inflammation [42]. More importantly, neutrophils are major constituents of the tumor microenvironment and determinants to potentiate macrophages recruitment and location in tumors [42–44]. We also found that upregulated KLK6 is correlated with macrophages enrichment of BLCA. Enrichment of tumor-associated macrophages contributes to chemotherapy resistance and correlates with poor prognosis [45]. Our findings suggest that KLK6 may participate in the carcinogenesis of BLCA via enriching tumor-associated immunocytes.

In conclusion, our results propose KLK6 as an independent prognostic factor and an antitumor target of BLCA. KLK6 expression positively correlates with several immune cells infiltration, indicating that inhibition of KLK6 may contribute to immunotherapy of BLCA.

## Data Availability

Not applicable.

## Abbreviations

KLK6: Kallikrein-related peptidase 6
GO: Gene ontology
BP: Biological process
MF: Molecular function
KEGG: Kyoto Encyclopedia of Genes and Genomes
TCGA: The Cancer Genome Atlas
BLCA: Bladder urothelial carcinoma
BCa: Bladder cancer.

## Supplementary Materials

Supplementary Materials Supplementary Figure 1. The association between KLK6 expression and KLK6 in pan-cancer. The association between KLK6 expression and KLK6 in pan-cancer was detected using the TCGA database. Supplementary Figure 2. After transfection with three siRNAs into RT4 and T24 for 72 h, cell growth was evaluated using CCK-8 assay. ^∗^*p* < 0.05, ^∗∗^*p* < 0.01, ^∗∗∗^*p* < 0.001 compared with si-Con group.
