# Supplementary material for: KLK6 Functions as an Oncogene and Unfavorable Prognostic Factor in Bladder Urothelial Carcinoma
Source: Dis Markers. 2022 Sep 22;2022:3373851. doi: 10.1155/2022/3373851 (PMC9526581; doi:10.1155/2022/3373851)
Supplement: Supplementary Materials — Supplementary Figure 1. The association between KLK6 expression and KLK6 in pan-cancer. The association between KLK6 expression and KLK6 in pan-cancer was detected using the TCGA database. Supplementary Figure 2. After transfection with three siRNAs into RT4 and T24 for 72 h, cell growth was evaluated using CCK-8 assay. ∗p < 0.05, ∗∗p < 0.01, ∗∗∗p < 0.001 compared with si-Con group. [file 3373851.f1.zip › supplementary figure 1.pdf]

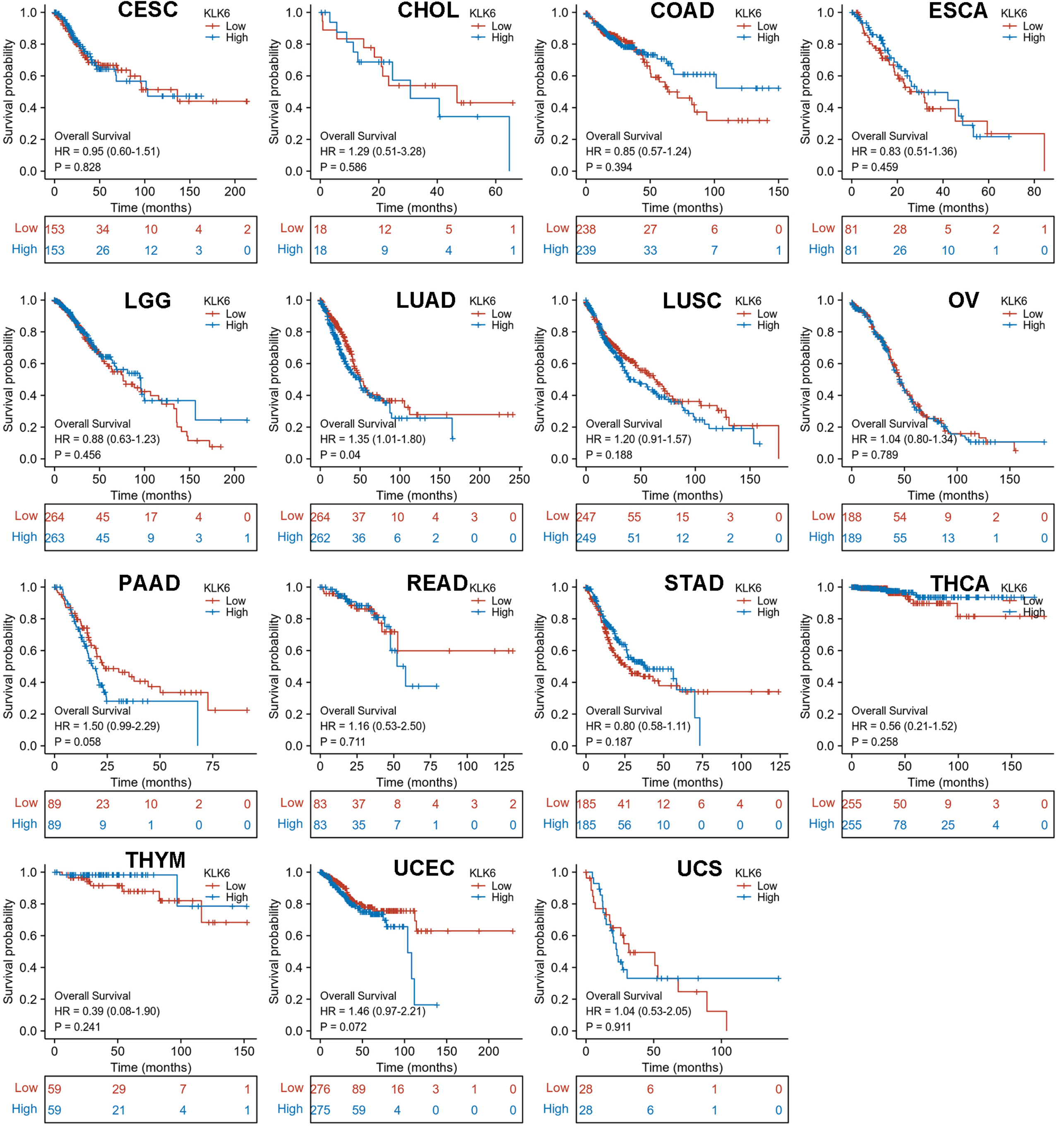

CESC
KLK6
Low
High
Overall Survival
HR = 0.95 (0.60-1.51)
P = 0.828
Survival probability
Time (months)
Low 153 34 10 4 2
High 153 26 12 3 0
CHOL
KLK6
Low
High
Overall Survival
HR = 1.29 (0.51-3.28)
P = 0.586
Survival probability
Time (months)
Low 18 12 5 1
High 18 9 4 1
COAD
KLK6
Low
High
Overall Survival
HR = 0.85 (0.57-1.24)
P = 0.394
Survival probability
Time (months)
Low 238 27 6 0
High 239 33 7 1
ESCA
KLK6
Low
High
Overall Survival
HR = 0.83 (0.51-1.36)
P = 0.459
Survival probability
Time (months)
Low 81 28 5 2 1
High 81 26 10 1 0
LGG
KLK6
Low
High
Overall Survival
HR = 0.88 (0.63-1.23)
P = 0.456
Survival probability
Time (months)
Low 264 45 17 4 0
High 263 45 9 3 1
LUAD
KLK6
Low
High
Overall Survival
HR = 1.35 (1.01-1.80)
P = 0.04
Survival probability
Time (months)
Low 264 37 10 4 3 0
High 262 36 6 2 0 0
LUSC
KLK6
Low
High
Overall Survival
HR = 1.20 (0.91-1.57)
P = 0.188
Survival probability
Time (months)
Low 247 55 15 3 0
High 249 51 12 2 0
OV
KLK6
Low
High
Overall Survival
HR = 1.04 (0.80-1.34)
P = 0.789
Survival probability
Time (months)
Low 188 54 9 2 0
High 189 55 13 1 0
PAAD
KLK6
Low
High
Overall Survival
HR = 1.50 (0.99-2.29)
P = 0.058
Survival probability
Time (months)
Low 89 23 10 2 0
High 89 9 1 0 0
READ
KLK6
Low
High
Overall Survival
HR = 1.16 (0.53-2.50)
P = 0.711
Survival probability
Time (months)
Low 83 37 8 4 3 2
High 83 35 7 1 0 0
STAD
KLK6
Low
High
Overall Survival
HR = 0.80 (0.58-1.11)
P = 0.187
Survival probability
Time (months)
Low 185 41 12 6 4 0
High 185 56 10 0 0 0
THCA
KLK6
Low
High
Overall Survival
HR = 0.56 (0.21-1.52)
P = 0.258
Survival probability
Time (months)
Low 255 50 9 3 0
High 255 78 25 4 0
THYM
KLK6
Low
High
Overall Survival
HR = 0.39 (0.08-1.90)
P = 0.241
Survival probability
Time (months)
Low 59 29 7 1
High 59 21 4 1
UCEC
KLK6
Low
High
Overall Survival
HR = 1.46 (0.97-2.21)
P = 0.072
Survival probability
Time (months)
Low 276 89 16 3 1 0
High 275 59 4 0 0 0
UCS
KLK6
Low
High
Overall Survival
HR = 1.04 (0.53-2.05)
P = 0.911
Survival probability
Time (months)
Low 28 6 1 0
High 28 6 1 0
