# Supplementary material for: KLK6 Functions as an Oncogene and Unfavorable Prognostic Factor in Bladder Urothelial Carcinoma
Source: Dis Markers. 2022 Sep 22;2022:3373851. doi: 10.1155/2022/3373851 (PMC9526581; doi:10.1155/2022/3373851)
Supplement: Supplementary Materials — Supplementary Figure 1. The association between KLK6 expression and KLK6 in pan-cancer. The association between KLK6 expression and KLK6 in pan-cancer was detected using the TCGA database. Supplementary Figure 2. After transfection with three siRNAs into RT4 and T24 for 72 h, cell growth was evaluated using CCK-8 assay. ∗p < 0.05, ∗∗p < 0.01, ∗∗∗p < 0.001 compared with si-Con group. [file 3373851.f1.zip › Supplementary figure legends.docx]

**Supplementary figure 1.** The association between KLK6 expression and KLK6 in Pan-cancer. The association between KLK6 expression and KLK6 in Pan-cancer was detected using the TCGA database.

**Supplementary figure 2.** After transfection with three siRNAs into RT4 and T24 for 72 h, cell growth was evaluated using CCK-8 assay. ^*^ p < 0.05, ^**^ p < 0.01, ^***^ p < 0.001 compared with si-Con group.
